# Syndromic ichthyoses

**DOI:** 10.1515/medgen-2023-2006

**Published:** 2023-04-05

**Authors:** Judith Fischer, Alrun Hotz, Katalin Komlosi

**Affiliations:** University of Freiburg Faculty of Medicine Freiburg Deutschland; University of Freiburg Faculty of Medicine Freiburg Deutschland; University of Freiburg Faculty of Medicine Freiburg Deutschland

**Keywords:** genodermatoses, Mendelian disorders of cornification, syndromic ichthyoses, inborn errors of metabolism

## Abstract

Inherited ichthyoses are classified as Mendelian disorders of cornification (MEDOC), which are further defined on the basis of clinical and genetic features and can be divided into non-syndromic and syndromic forms. To date, mutations in more than 30 genes are known to result in various types of syndromic ichthyoses, which, in addition to mostly generalised scaling and hyperkeratosis of the skin, also show additional organ involvement. The syndromic ichthyoses are generally very rare and are classified based on the mode of inheritance, and can be further subdivided according to the predominant symptoms.

In our review we provide a concise overview of the most prevalent syndromic forms of ichthyosis within each subgroup. We emphasize the importance of the clinical assessment of complex syndromes even in the era of genetic testing as a first-tier diagnostic and specifically the need to actively assess potential organ involvement in patients with ichthyosis, thereby enabling efficient diagnostic and therapeutic approaches and timely access to specialized centers for rare disorders of cornifications. As part of the Freiburg Center for Rare Diseases a Center for Cornification Disorders was recently established with collaboration of the Institute of Human Genetics and the Department of Dermatology.

An early diagnosis of syndromes will be of direct benefit to the patient regarding interventional and therapeutic measures e. g. in syndromes with cardiac or metabolic involvement and allows informed reproductive options and access to prenatal and preimplantation genetic diagnosis in the family.

## Introduction

The syndromic ichthyoses are cornification disorders which, in addition to mostly generalised scaling and hyperkeratosis of the skin, also show additional symptoms in other organ systems. They are therefore not limited to the skin.

The clinical diagnosis of syndromic ichthyoses can be greatly facilitated by recognising the additional characteristic symptoms that are present. Therefore, knowledge of the specific constellation of symptoms in the respective syndromic forms of ichthyosis can contribute to the successful establishment of a clinical diagnosis.

Syndromic ichthyoses can be divided into X-linked and autosomal according to the mode of inheritance. The first group includes syndromic X-linked recessive ichthyosis (XRI), the XR-IFAP syndrome and X-linked dominant (XD) chondrodysplasia punctata. The second group includes all other syndromic cornification disorders that follow an autosomal recessive or dominant inheritance and are further subdivided according to the predominant accompanying symptoms (Table 1).

In the following we have selected some typical and relevant examples for a more detailed description.

## 1 X-chromosomal inherited syndromic ichthyoses

### 1.1 Syndromic X-linked recessive ichthyosis

**Fig. 1:**
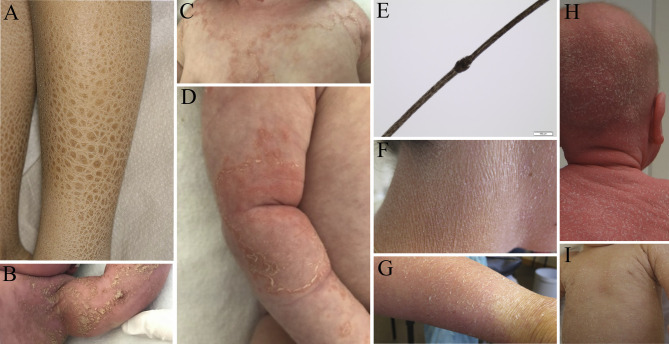
(A) rhomboid, dark-brown scaling on the lower legs in X-linked recessive ichthyosis (Courtesy of Dr. Emmanuelle Bourrat, Paris, France); (B) inflammatory phenotype with pronounced feather-like scaling and hyperkeratosis in Conradi-Hünermann-Happle syndrome (Courtesy of Dr. Nicola Dikow, Heidelberg, Germany); (C, D,E) Netherton syndrome: (C, D) serpiginous and migratory erythematous patches with double-edged scales (ichthyosis linearis circumflexa) (Courtesy of Dr. Lothar Schrod and Dr. Petra Ley, Frankfurt, Germany), (E) light microscope image of a bamboo hair (trichorrhexis invaginata) (Dr. Ingrid Hausser-Siller); (F, G) generalized ichthyosiform erythroderma and scaling in Chanarin-Dorfman syndrome (Prof. Angelika Rieß, Tübingen, Germany); (H) severe generalized ichthyosiform erythroderma with hypotrichosis in SAM syndrome and (I) mild to moderate generalized ichthyosiform erythroderma in KIDAR syndrome (Prof. Christoph Korenke, Oldenburg, Germany, and Prof. Hagen Ott, Hannover, Germany)

While partly deletions of the *STS* gene (90 %) or rarely point mutations lead to non-syndromic XRI with isolated involvement of the skin, larger deletions within Xp22.3 often involve multiple adjacent genes, leading to a contiguous gene deletion syndrome. Besides ichthyosis (Figure 1 A), the additional symptoms depend on the extent of the deletion and may involve intellectual disability, hypogonadotrophic hypogonadism and anosmia in Kallmann syndrome, microcephaly, hearing loss, cataracts and skeletal abnormalities in X-linked chondrodysplasia punctata, short stature in SHOX deficiency disorder and ocular albinism [1, 2, 3]. Recently it was shown that abnormal heart rhythms may affect up to 35 % of Xp22.31 deletion carriers, show no consistent pattern of onset but may be precipitated by stress, and typically resolve quickly and respond well to intervention [4].

### 1.2 IFAP syndrome-1

The IFAP syndrome-1 with or without BRESHECK syndrome is a X-linked multiple congenital anomaly disorder with variable organ involvement caused by hemizygous or heterozygous mutations in the *MBTPS2* gene on chromosome Xp22. Recently, an autosomal dominant form of IFAP syndrome due to heterozygous mutations in *SREBF1* has been described [5]. The classic triad involves **i**chthyosis **f**ollicularis, **a**trichia, and **p**hotophobia and was first described by MacLeod in l909 [6]. Some patients show additional features including intellectual disability, structural brain anomalies, Hirschsprung disease, corneal opacifications, kidney dysplasia, cryptorchidism, cleft palate, and skeletal malformations, particularly of the vertebrae, which constitutes BRESHECK syndrome [7]. Recently, cytopenia, bone marrow fibrosis and chronic diarrhea were described as additional features [8]. Female carriers can sometimes present minimal symptoms, such as an asymmetrical distribution of body hair, patchy alopecia or hyperkeratosis along the Blaschko lines. In addition to the absence of scalp hair, eyebrows and eyelashes, the complete atrichia of body hair is part of the full spectrum of IFAP syndrome in male patients.

### 1.3 Conradi-Hünermann-Happle syndrome

Also known as chondrodysplasia punctata 2 (CDPX2) Conradi-Hünermann-Happle syndrome is one of the XD disorders that are lethal in male fetuses. Exceptions are possible in postzygotic mosaics or in Klinefelter syndrome (47,XXY). The characteristic symptoms of female patients include asymmetrical punctiform calcification of the bones, sectoral cataracts, and streaky skin changes following the Blaschko lines. In addition to radiographic stippling, the disorder is characterized by rhizomelic shortness, transient congenital ichthyosis following the lines of Blaschko, patchy alopecia, cataracts, and midface hypoplasia. The stippling chondrodysplasia punctata is visible as a splashed paint pattern [9] in the X-ray picture until approximately the ninth month of life. In the first few weeks of life, there is a very inflammatory phenotype with pronounced feather-like scaling and hyperkeratosis (Figure 1 B), which later turns into follicular atrophoderma [10]. CDPX2 is caused by mutations in the *EBP* gene, which codes for the delta(8)-delta(7) sterol isomerase on chromosome Xp11, also known as emopamil binding protein (EBP), involved in cholesterol metabolism [3, 11].

## 2 Autosomal inherited syndromic ichthyoses with hair anomalies

### 2.1 Netherton syndrome

Netherton syndrome (NS) is an autosomal recessive disorder and clinically characterized by congenital ichthyosiform erythroderma, trichorrhexis invaginata, and atopic diathesis. Affected children present a generalized ichthyosiform erythroderma at birth, which can persist later in life, or develop into ichthyosis linearis circumflexa, which presents as serpiginous and migratory erythematous patches with double-edged scales (Figure 1 C, D). Children often show severe growth and developmental delay, which is partly caused by diarrhea, intestinal malabsorption, recurrent infections and hypernatraemic dehydration. Trichorrexis invaginata is also known as Bamboo hair and can be diagnosed by dermatoscopy of the hair (Figure 1 E). This hair anomaly is pathognomonic for NS and can be used as a confirmation of the diagnosis. Affected individuals show brittle hair and poor hair growth. Patients with NS typically show different atopic manifestations, increased serum IgE, and hypereosinophilia. However, not all patients with NS show the entire spectrum of the possible symptoms. NS is caused by biallelic pathogenic variants in the *SPINK5* gene which encodes the serine protease inhibitor LEKTI (Lympho-epithelial Kazal-type-related inhibitor). The disruption of LEKTI leads to inflammatory processes in the epidermis. Treatment with immunoglobulins and biologicals shows good results in NS patients [12].

## 3 Autosomal inherited syndromic ichthyoses with neurologic involvement

### 3.1 Sjögren-Larsson syndrome

The Sjögren-Larsson syndrome (SLS) was first reported in 1957 by Swedish physicians [13]. The SLS is an autosomal recessive neurocutaneous disorder and characterized by the triad of congenital ichthyosis, spasticity, and mental retardation. Many affected infants were born prematurely. After birth, they show reddened skin with fine scales, which generally progresses to generalized brown ichthyosis, especially in the neck, lower abdomen, extremities and articular folds, often accompanied by mild to severe itchiness. Most affected individuals show intellectual disability of different degrees, dysarthria and speech delay. Imaging with MRI often shows leukencephalopathy in affected patients [14]. A characteristic sign of SLS is a delayed development of motor skills due to progressive spasticity in the legs and, less commonly, also in the arms. Many patients never learn to work independently [15]. Possible additional symptoms are epilepsy, short stature, scoliosis, neuropathy and ophthalmological abnormalities. In rare cases, the phenotype can be very mild [16]. The causative gene for SLS is *ALDH3A2*, which encodes the fatty aldehyde dehydrogenase (FALDH). The FALDH enzyme is important for the catalyzation the fatty acid oxidation [17]. Currently, there is no curative therapy for SLS. However, there are some prospective therapeutic options which are targeted to specific metabolic defects associated with FALDH deficiency, such as fatty aldehyde scavengers [18].

### 3.2 MEDNIK syndrome

MEDNIK syndrome is a rare autosomal recessive severe neuro-cutaneous disease. It is characterized by **m**ental retardation, **e**nteropathy, **d**eafness, **n**europathy, **i**chthyosis and **k**eratoderma [19]. Affected patients show a copper overload, which leads to an accumulation of copper in the liver. The copper overload can be treated with zinc acetate to reduce copper intestinal absorption. This improves the clinical and neurological conditions [20]. Imaging with MRI generally shows cerebral atrophy in affected individuals. MEDNIK syndrome is caused by biallelic pathogenic mutations in *AP1S1*. This gene codes for the sigma-1A subunit of adaptor protein complex 1 that directs intracellular trafficking of copper pumps ATP7A and ATP7B [21].

## 4 Autosomal inherited syndromic ichthyoses with hearing loss

### 4.1 KID syndrome

The KID syndrome is an autosomal dominant inherited disorder characterized by keratitis, ichthyosis and deafness. Affected individuals generally show a very pronounced erythrokeratoderma. Additional symptoms may be progressive erythematous dermatitis, palmoplantar keratoderma, nail dystrophy, abnormal teeth, sparse head hair, eyebrows and eyelashes. There is an increased risk for developing squamous cell carcinoma of the skin or mucous membranes. Affected individuals mainly develop keratitis, which can result in photophobia, neovascularization and progressive visual deterioration. Most affected individuals present profound hearing loss, less often slight hearing loss. Patients with KID syndrome have an increases risk for the development of skin cancer [22]. The main causative gene for KID syndrome is the gap junction protein beta 2, *GJB2*, which encodes the structural protein connexin 26. This protein forms gap junction channels between neighboring cells. The impairment of these channels affect direct communication from cell to cell in the skin, cornea and inner ear. Pathogenic variants in *GJB2* can also lead to autosomal recessive inherited hearing loss or autosomal dominant inherited palmoplantar keratoderma type Vohwinkel. In very rare cases, KID syndrome can be caused by mutations in the gap junction protein beta 6, *GJB6* (connexin 30) [23].

### 4.2 ARKID syndrome

The acronym ARKID means **a**utosomal **r**ecessive **k**eratoderma, **i**chthyosis and **d**eafness. The ARKID syndrome is caused by biallelic mutations in *VPS33B* [24]. Pathogenic mutations in *VPS33B* are also known as causative for arthrogryposis, renal dysfunction and cholestasis (ARC syndrome, see below). Possible additional symptoms in ARKID syndrome besides those mentioned above are hearing loss, intellectual disability, hip dislocation, microcephaly, short stature, and copper overload, which is also known in MEDNIK syndrome [25].

### 4.3 KIDAR syndrome

The autosomal recessive keratitis-ichthyosis-deafness syndrome (KIDAR) is caused by biallelic pathogenic mutations in *AP1B1* [26, 27]. Affected individuals can present ichthyosis (Figure 1 I), erythroderma and palmoplantar keratoderma. Keratitis and corneal scarring can lead to photophobia and vision loss. Progressive hearing loss and developmental delay are often seen in affected persons [28]. Similar to MEDNIK syndrome, there is an abnormal copper metabolism in KIDAR syndrome [29]. *AP1B1* encodes the beta-1A subunit of adaptor protein complex 1. A disruption leads to a defect in the function of AP-1, which is important for normal copper transporters. The pathogenesis of both MEDNIK and KIDAR syndrome affect the same pathway of regulation of AP-1 which contributes to the trafficking of transmembrane proteins.

## 5 Autosomal inherited syndromic ichthyoses with fatal disease progression

### 5.1 CEDNIK syndrome

The acronym CEDNIK syndrome refers to the typical constellation of **ce**rebral **d**ysgenesis, **n**europathy, **i**chthyosis and palmoplantar **k**eratoderma but not all the symptoms are always present [30]. This rare AR progressive neurocutaneous disease is caused by loss-of-function variants of the *SNAP29* gene encoding the synaptosomal-associated protein 29, a member of the SNARE family of proteins that is involved in the intracellular transport in various vesicle and membrane fusion processes [31]. Patients with CEDNIK syndrome typically present with ichthyosis, palmoplantar keratoderma, intellectual disability, microcephaly, brain malformations such as corpus callosum dysgenesis and cortical dysplasia with pachygyria and polymicrogyria, facial dysmorphism, hypoplastic optic disks, sensorineural deafness, and failure to thrive, however, a recent review of 25 cases showed considerable phenotypic variability [32].

### 5.2 ARC syndrome

**A**rthrogryposis-**r**enal dysfunction-**c**holestasis syndrome is clinically characterized by the association of arthrogryposis, renal tubular abnormalities, cholestasis, hypotonia and severe failure to thrive. Patients with this AR inherited multisystem disorder also develop severe ichthyosis in addition to deafness, platelet abnormalities, osteopenia, absent corpus callosum, recurrent infections and facial dysmorphism. Most affected children die early [3, 33]. ARC syndrome 1 (ARCS1) is caused by mutations in the *VPS33B* gene [34]. ARCS2 is caused by biallelic mutations in *VIPAS39* [35]. Both genes play an important role in the biogenesis and function of lamellar bodies in the epidermis [36].

## 6 Ichthyosis associated with inborn errors of metabolism

### 6.1 Congenital disorders of glycosylation associated with ichthyosis

Rare forms of congenital disorders of glycosylation (CDG) are among the inborn errors of metabolism presenting at birth with ichthyosis [3]. CDG are due to deficient glycosylation of proteins and lipids and are characterized by multi-organ symptoms with a wide range of clinical severity, from mild symptoms, to severe multisystem dysfunction, and even a fatal course [37]. The spectrum of clinical manifestations comprises psychomotor delay and intellectual disability, muscle hypotonia, seizures, endocrine and coagulation abnormalities, ophthalmologic anomalies, failure to thrive and variable dysmorphic features. Skin abnormalities are described in about 20 % of the different CDG forms [38, 39, 40, 41]. CDG linked to ichthyosis or ichthyosiform dry skin with variable neurologic and multi-organ involvement are due to deficiencies within the dolichol (DOLK‐, SRD5A3‐CDG) and the GPI anchor biosynthesis pathway (PIGL‐CDG, MPDU1‐CDG) [38, 39, 42, 43, 44, 45, 46, 47, 48].

**Dolichol kinase deficiency** **(DOLK-CDG)** is an autosomal recessive disorder that results from deficiency of the enzyme responsible for the terminal step in the synthesis of dolichol phosphate. The phenotypic spectrum encompasses three major forms with 1. Neurological abnormalities with hypotonia, microcephaly, seizures, visual impairment with or without ichthyosis, 2. Isolated cardiomyopathy and 3. Multi-organ involvement, which can also include ichthyosis and lead to death within the first months of life [43, 46, 48]. We recently described siblings presenting at birth with severe ichthyosis, distal digital constrictions, cardiomegaly, thrombocytopenia, diffuse coagulation defect and progressive multi-organ failure, which resulted in death in the neonatal period [41].

**SRD5A3-CDG (cerebro-cerebello-oculo-cutaneous syndrome)** is due to the deficiency of polyprenol reductase within the biosynthesis of dolichol. Patients present with loose skin at birth, ichthyosiform dermatitis, hyperkeratosis, particularly on palms and soles of feet and dark pigmentation of knees and dorsum of hands and feet. Additionally, patients may present coloboma and optic disc atrophy with loss of vision, intellectual disability, cerebellar malformations, and coagulation defects [49].

**MPDU1-CDG** is a defect in the N-glycan assembly in the endoplasmic reticulum (ER) [42, 47]. Patients present skin symptoms (ichthyosis, erythroderma), neurological features (psychomotor retardation, seizures), hypotonia, visual impairment, growth deficiency due to transient growth hormone deficiency.

Patients with **PIGL-CDG or CHIME syndrome** (or Zunich neuroectodermal syndrome) mainly have colobomas, congenital heart defects, early-onset migratory ichthyosiform dermatosis, intellectual disability, and ear anomalies. The defect is localized in the ER and concerns the second step of the GPI anchor biosynthesis pathway [45].

Mild skin manifestations were also observed in a patient with **COG5‐CDG** [50] and recently we described a family with two affected children with **COG6-CDG** in whom very dry, tight and rigid skin with hyperkeratosis and scaling was the prominent skin feature at birth [40].

The above mentioned rare CDG forms emphasize the phenotypic variability of glycosylation disorders and the importance to broaden the differential diagnosis of ichthyosis and to actively search for organ involvement in neonates with ichthyosis.

### 6.2 Lysosomal storage disorders associated with ichthyosis

Some lysosomal storage disorders present, in addition to variable organ involvement, also a more or less pronounced ichthyosis. The AR **multiple sulfatase deficiency (MSD)** is caused by mutations in the *SUMF1* gene (sulfatase-modifying factor 1) [51] resulting in tissue accumulation of sulfatides, sulfated glycosaminoglycans, sphingolipids, and steroid sulfates. The enzymatic defect affects the whole family of sulfatase enzymes, thus, combining features of metachromatic leukodystrophy and of various mucopolysaccharidoses. Affected individuals show neurologic deterioration with intellectual disability, skeletal anomalies, organomegaly, and ichthyosis [52]. Different types of MSD can be distinguished according to the age of onset: neonatal, late infantile (0 to 2 years), and juvenile (2 to 4 years). Neonatal MSD is the most severe form with a broad range of mucopolysaccharidosis-like symptoms and death within the first year of life. The diagnosis can be established biochemically by detecting increased simultaneous excretion of heparan sulfate, dermatan sulfate, keratan sulfate and chondroitin sulfate and sulfatides and confirmed by biallelic mutations in the *SUMF1* gene.

**Gaucher disease type 2 (GD2)**. The presence of ichthyosis or collodion membrane at birth has only been observed in the rare type 2 Gaucher disease [53]. The further course of the disease is fatal, due to the occurrence of hepatosplenomegaly and progressive neurological symptoms, such as spasticity, seizures and oculomotor paralysis. A recent review found that GD2 may present prenatally with hydrops fetalis, in the newborn period, or later in the first year of life. In the newborn period, patients may present with congenital ichthyosis, hepatosplenomegaly, biliary atresia, facial dysmorphology, arthrogryposis, congenital thrombocytopenia, and/or growth abnormalities. The ichthyosis may result from increased glucosylceramide in the stratum corneum, which leads to abnormal histologic appearance of the skin. Patients presenting later in the infantile period may show failure to thrive, swallowing abnormalities, seizures, developmental delay, and/or abnormal eye movements [54]. Affected children usually die before their second year of life. The disease is caused by biallelic mutations in the *GBA* gene, which encodes the lysosomal enzyme acid beta-glucosidase (or beta-glucocerebrosidase), and plays a role in ceramide metabolism.

## 7 Syndromic ichthyoses with other symptoms

### 7.1 Chanarin-Dorfman syndrome

The Chanarin-Dorfman syndrome is an autosomal recessive inherited neutral lipid storage disease with ichthyosis (NLSDI) caused by mutations in *ABHD5* [55]. This gene encodes the protein CGI-58, which interacts with adipose triacylglycerol lipase (ATGL), also known as PNPLA2. The enzymatic function is the breakdown of triglycerides in the cell. The disturbation of this process leads to an accumulation of lipid vacuoles in many cell types. One simple diagnostic method is a peripheral blood smear, in which the lipid droplets can be seen in granulocytes. This phenomenon is known as Jordan anomaly. Lipid vacuoles in granulocytes also appear in another lipid storage disorder with severe myopathy, but no ichthyosis (NLSDM). This disorder is caused by mutations in *PNPLA2* [56]. Patients with Chanarin-Dorfman syndrome are often born as collodion babies and show mild generalized ichthyosiform erythroderma (Figure 1F, G) later in life, often accompanied by intense itching. The accumulation of lipid vacuoles in many cell types explain the wide range of additional symptoms. In the liver the development of steatosis is frequent, which can progress to cirrhosis and liver failure. The skeletal musculature can be affected with muscle weakness and myopathy with increasing age. Furthermore, cardiomyopathy can occur in some patients. Additional symptoms such as hearing loss, visual impairment, ataxia and mental retardation may occur less frequently.

### 7.2 SAM syndrome

The presence of eczema and elevated IgE in pediatric patients is not exclusively indicative of atopic dermatitis. Rare genodermatoses may present a similar or even more severe clinical picture and should be considered in the differential diagnosis of congenital immunodeficiency and severe refractory dermatitis. Among the syndromic ichthyosis the SAM syndrome belongs to the inherited disorders of desmosomes [57]. The cohesion between keratinocytes ensures the integrity of the epidermis, and desmosomes represent the main adhesion structures. During cornification desmosomes are modified and transformed into corneodesmosomes. Mutations in the genes coding for desmosomal components lead to several skin diseases including palmoplantar keratoderma, certain forms of epidermolysis bullosa, SAM syndrome and ectodermal dysplasia-skin fragility (EDSF) syndrome (Table 1). SAM syndrome is characterized by 3 predominant symptoms: **s**evere dermatitis, **m**ultiple allergies and **m**etabolic wasting. The phenotype involves generalised erythroderma with severe itching, hypotrichosis (Figure 1 H), enamel defects, onychodystrophy, palmoplantar keratoderma, failure to thrive and recurrent infections. Further characteristics are massively elevated levels of IgE, as in Netherton syndrome and allergies to multiple allergens due to severe barrier impairment [57, 58, 59].

Initially, patients with biallelic loss-of function mutations in the desmoglein 1 (*DSG1*) gene with an AR inheritance pattern were described [57, 58]. Later, cases with SAM syndrome were also diagnosed with monoallelic desmoplakin (*DSP*) [59] and most recently, with monoallelic *DSG1* mutations. Precise genotype-phenotype correlations were demonstrated for *DSG1* mutations [60] and a recent study elucidating the underlying pathomechanism suggested that heterozygous dominant negative mutations in the transmembrane domain (TMD) of *DSG1* lead to AD SAM syndrome, whereas heterozygous loss-of-function mutations in the TMD cause the milder PPK disease phenotype [61].

## Conclusion

An early diagnosis of syndromes is equally important for patients, their families and the treating physicians. As one of the most important and direct benefits to the patient, interventional and therapeutic measures can be taken e. g. in syndromes with cardiac or metabolic involvement, thus counteracting possible life-threatening situations. In regard to the families a genetically confirmed diagnosis is essential for informed reproductive options and access to prenatal and preimplantation genetic diagnosis.

**Tab. 1:** Syndromic ichthyoses (Table adapted from Fischer and Bourrat, 2020)

**Name**	**Abbreviation**	**OMIM**	**Mode of inheritance**	**Gene/Locus**
**X-chromosomal inherited syndromes**				
Syndromic XR ichthyosis^1^	XRI	308100	XR	Xp22-deletion^2^
IFAP syndrome	IFAP	308205	XR	*MBTPS2*
Conradi-Hünermann-Happle syndrome	CDPX2	302960	XD	*EBP*
**Autosomal inherited syndromes with:**				
**Hair anomalies**				
Comèl-Netherton syndrome	NS	256500	AR	*SPINK5*
Ichthyosis-hypotrichosis syndrome	IHS	602400	AR	*ST14*
IHCS syndrome	IHCS	607626	AR	*CLDN1*
Trichothiodystrophie (photosensitive)	TTDP	601675	AR	*ERCC2/XPD, ERCC3/XPB, GTF2H5/TTDA*
**Prominent neurological symptoms**				
Sjögren-Larsson syndrome Refsum syndrome	SLS RS	270200 266500	AR AR	*ALDH3A2* *PHYH, PEX7*
MEDNIK syndrome	MEDNIK	609313	AR	*AP1S1*
IKSHD (*ELOVL1*-deficit)	IKSHD	618527	AD	*ELOVL1*
**Hearing loss**				
KID syndrome ARKID syndrome KIDAR syndrome	KID ARKID KIDAR	148210 620009 242150	AD AR AR	*GJB2, GJB6* *VPS33B* *AP1B1*
**Fatal disease progression**				
CEDNIK syndrome	CEDNIK	609528	AR	*SNAP29*
ARC syndrome	ARC	208085	AR	*VPS33B*
**Inborn errors of metabolism** **Congenital disorders of glycolysation (CDG)** Dolichol kinase deficiency Cerebro-cerebello-oculo-cutaneous syndrome MPDU1-CDG CHIME syndrome COG5-CDG COG6-CDG	DOLK-CDG SRD5A3-CDG MPDU1-CDG PIGL-CDG COG5-CDG COG6-CDG	610768 612379 609180 280000 613612 614576	AR AR AR AR AR AR	*DOLK* *SRD5A3* *MPDU1* *PIGL* *COG5* *COG6*
**Lysosomal storage disorders (LSD)** Multiple Sulfatase Deficiency Gaucher disease type 2	MSD GS	272200 230900	AR AR	*SUMF1* *GBA*
**Other inborn errors of metabolism** Phosphoserine aminotransferase deficiency/ Neu-Laxova syndrome 2 Neu-Laxova syndrome 1 Holocarboxylase synthetase deficiency Transaldolase deficiency	PSATD NLS1 HLCSD TALDOD	610992 256520 253270 606003	AR AR AR AR	*PSAT1* *PHGDH* *HLCS* *TALDO1*
**Other symptoms**				
Chanarin-Dorfman syndrome	NLSDI/ CDS	275630	AR	*ABHD5/CGI-58*
SAM syndrome Ectodermal dysplasia-skin fragility syndrome HELIX syndrome IFAP syndrome 2	SAM EDSF HELIX IFAP2	615508 604536 617671 619016	AR/AD AR AR AD	*DSG1, DSP* *PKP1* *CLDN10* *SREBF1*

^1^Symptoms depending on the size of the deletion. ^2^contiguous gene deletion syndrome: *STS* and other genes may be deleted. P**revalence** of the syndromic ichthyosis: the **syndromic XR ichthyosis** has a prevalence of **1-9/100 000**, all other forms of syndromic ichthyoses are estimated to have a **prevalence of 1-9/1 000 000 or <1/ 1 000 000** (Orphanet).

**Abbreviations**: XR X-linked recessive; XD X-linked dominant; AR autosomal recessive; AD autosomal dominant; IFAP ichthyosis follicularis-atrichia-photophobia; CDPX2 chondrodysplasia punctata 2; IHSC ichthyosis hypotrichosis sclerosing cholangitis; MEDNIK Mental retardation-enteropathy-deafness-neuropathy-ichthyosis-keratoderma; IKSHD ichthyosis, keratoderma, spasticity, hypomyelination, dysmorphia; KID keratitis-ichthyosis-deafness; ARKID Autosomal recessive keratoderma-ichthyosis-deafness; KIDAR keratitis-ichthyosis-deafness syndrome; CEDNIK Cerebral dysgenesis-neuropathy-ichthyosis-palmoplantar keratoderma; ARC arthrogryposis-renal dysfunction-cholestasis; CHIME syndrome coloboma, congenital heart disease, ichthyosiform dermatosis, mental retardation, ear anomalies syndrome; NLSDI Neutral lipid storage disease with ichthyosis; SAM Severe dermatitis, multiple allergies and metabolic wasting; HELIX hypohidrosis, electrolyte imbalance, lacrimal gland dysfunction, ichthyosis, xerostomia

Even in times when whole-exome sequencing or whole genome sequencing is increasingly used as a first-tier diagnostic method, it remains an essential medical task to be able to clinically assess the syndromes in their complexity and to draw the right conclusions for further diagnostic and therapeutic procedures.

The clinical diagnosis of syndromic ichthyoses is aided in many cases through the easy access to the clinical examination of the skin, mucosa and skin appendages such as hair, nails and teeth and the typical constellation of extracutaneous symptoms). However, in many cases it is important to search for specific and less obvious symptoms. Furthermore, the large heterogeneity of syndromic ichthyoses can make the clinical diagnosis challenging. In more than 30 syndromes with ichthyoses, the molecular genetic background is known and the accessibility of genetic testing enables a definitive diagnosis in an increasing number of cases. With the widespread use of broad diagnostic methods, further, more rarely occurring types will be resolved in the future.
